# Preparation and Optimization of Waterborne Acrylic Core Microcapsules for Waterborne Wood Coatings and Comparison with Epoxy Resin Core

**DOI:** 10.3390/polym12102366

**Published:** 2020-10-15

**Authors:** Xiaoxing Yan, Yu Tao, Xingyu Qian

**Affiliations:** 1 Co-Innovation Center of Efficient Processing and Utilization of Forest Resources, Nanjing Forestry University, Nanjing 210037, China; 2 College of Furnishings and Industrial Design, Nanjing Forestry University, Nanjing 210037, China; taoyu@njfu.edu.cn (Y.T.); qianxingyu@njfu.edu.cn (X.Q.)

**Keywords:** waterborne acrylic resin, microcapsule, waterborne coating

## Abstract

Microcapsules were prepared by in situ polymerization with urea formaldehyde resin as the wall material and Dulux waterborne acrylic acid as the core material. The effects of the core–wall ratio, water bath temperature and depositing time on the morphology, particle size, yield and encapsulation ratio of microcapsules were investigated by orthogonal experiment of three factors and two levels. The results showed that the core–wall ratio had the greatest influence on the performance of microcapsules. When the core–wall ratio was 0.58:1, the water bath temperature was 70 °C, and the depositing time was 5 d, the microcapsule performance was the best. With the increase in depositing time, the yield of microcapsule particles increased gradually, and the microcapsules appeared to show an adhesive phenomenon. However, the long-term depositing time did not lead to complete deposition and agglomeration of microcapsules. When 10.0% concentration of the waterborne acrylic microcapsules with 0.58:1 of core–wall ratio was added to the coatings, the mechanical and optical properties of the coatings did not decrease significantly, but the elongation at break increased significantly. Therefore, this study offers a new prospect for using waterborne acrylic microcapsules to improve the toughness of waterborne paint film which can be cured at room temperature on a wood surface.

## 1. Introduction

Microcapsule technology is a kind of packaging technology that uses natural or synthetic polymer film-forming materials to coat gas, liquid or solid into 1–1000-μm micro particles [1]. Microcapsules are generally composed of a wall material (wrapping material) and core material (wrapped material). Due to the difference in the preparation methods and product requirements, the microcapsules have a variety of structures. The wall material determines the strength of microcapsules, the release characteristics of sustained-release core materials and the durability of microcapsules, which has an important impact on the performance of microcapsules [2]. Microencapsulation technology has experienced a long development process, and has been widely used in the fields of medical treatment [3], cosmetics, food, textile, coating and advanced materials. Although its application in wood coatings is still in the development stage, it has great application prospects [4,5,6]. Self-healing technology, as a new effective way to suppress coating micro-cracks, has become a research hotspot in many application fields [7,8,9]. Functionalized and application-oriented microcapsules are added to the coating to improve the repair ratio of the coating while enhancing the coating performance [10]. The choice of microcapsule wall material and core material seriously affects the performance of microcapsules [11,12].

Mirabedini et al. [13] applied a phase separation to prepare new oil-filled microcapsules with ethyl cellulose as the shell material, and investigated the relationship between the mass fraction of microcapsules and the tensile properties of the film. The results showed that the film had self-healing properties after adding microcapsules. Safaei et al. [14] used microcapsules containing epoxy healing agents to develop economic and efficient self-healing epoxy coatings. The effects of the preparation process on the properties of microcapsules were studied, and optimal synthesis conditions were obtained. Wang et al. [15] prepared microcapsules by in situ polymerization using poly(melamine-formaldehyde) as the wall materials and the mixture of bisphenol A diglycidyl ether and epoxy diluent as the core materials. The influences of epoxy diluent type, kinds of surfactant, mass fraction of emulsifier, and emulsification ratio on the physical performance of microcapsules were discussed. Zhang et al. [16] synthesized polyurea formaldehyde microcapsules with tall oil fatty acid poxy ester as core materials by in situ polymerization. The self-healing coatings were prepared by adding the polyuria formaldehyde epoxy ester microcapsules into the epoxy coatings. As a common type of waterborne coating, waterborne acrylic coatings are widely used [17]. The waterborne acrylic resin is a one-component repair agent that does not require a curing agent and a catalyst [18,19]. At present, there are many studies on the preparation of self-healing microcapsules using epoxy repair agents as the core material, while the research on the preparation of self-healing microcapsules for waterborne coatings with waterborne paint components as the core material is less extensive [20,21].

In this paper, microcapsules with urea-formaldehyde resin as the wall material and Dulux waterborne acrylic resin [22,23] as the core material were prepared by in situ polymerization. It was assumed that morphology, particle size, yield and encapsulation ratio of microcapsules had no relationship with the core–wall ratio. If the hypothesis was not true, it was considered that the performance of microcapsules was related to the core–wall ratio. The method of testing was proved by orthogonal experiment. Combined with the orthogonal test results, the preparation process of microcapsules was further optimized to determine the better morphology, yield and encapsulation performance of Dulux waterborne acrylic microcapsules, which provides a technical reference for the increased toughness of waterborne paint film dried at room temperature on the surface of the wood.

## 2. Materials and Methods 

### 2.1. Materials

The 37.0% formaldehyde solution (*M*_w_: 30.03 g/mol, CAS No.: 50-00-0), urea (*M*_w_: 60.06 g/mol, CAS No.: 57-13-6), triethanolamine (*M*_w_: 149.19 g/mol, CAS No.: 102-71-6), and ethyl acetate (*M*_w_: 88.11 g/mol, CAS No.: 141-78-6) were provided by Nanjing Chemical Reagent Co., Ltd., Nanjing, China. Dulux waterborne acrylic resin was offered by Akzo Nobel Paints (Shanghai) Co., Ltd., Shanghai, China. Sodium dodecyl benzene sulfonate (*M*_w_: 348.48 g/mol, CAS No.: 25155-30-0) was supplied by Beichen District Fangzheng Reagent Factory, Tianjin, China. Octanol (*M*_w_: 130.23 g/mol, CAS No.: 111-87-5) was offered by Yatai United Chemical Co., Ltd., Wuxi, China. Citric acid (*M*_w_: 210.14 g/mol, CAS No.: 5949-29-1) was provided by Beilian Fine Chemical Development Co., Ltd., Tianjin, China. Ethanol (*M*_w_: 46.07 g/mol, CAS No.: 64-17-5) was provided by Outuopu Biotechnology Co., Ltd., Hangzhou, China. Tilia europaea (100 mm × 65 mm × 4 mm, color uniformity, after sanding pretreatment) was offered by Yihua Lifestyle Technology Co., Ltd., Guangdong, China.

### 2.2. Preparation of Microcapsules

The three-factor two-level orthogonal test was used to determine the factor that had the greatest impact on the performance of the microcapsules. Then, the single-factor independent test was conducted on the factor that had the greatest influence. In order to explore the most important factors affecting the morphology, particle size, yield and encapsulation ratio of microcapsules [24], two levels of core–wall ratio, water bath temperature and depositing time were selected to conduct orthogonal experiments. The data of the orthogonal experiment arrangement are shown in Table 1.

The preparation process of urea-formaldehyde, resin-coated waterborne acrylic microcapsules mainly includes three parts: preparation of wall material urea-formaldehyde, preparation of core material emulsion and microencapsulation. The first step was the preparation of the wall material: 20.0 g of urea and 27.0 g of 37.0% formaldehyde solution were added to the beaker and fully stirred with a magnetic stirrer at 100 rpm. After the urea was completely dissolved, the triethanolamine was laxly mixed to regulate the pH value to about 9.0. The mixed solution was then put in a 70 °C water bath and continuously stirred for 90 min to obtain a slightly viscous and transparent urea-formaldehyde prepolymer solution. The solution was cooled to room temperature for use. The second step was the preparation of the core material: the 0.975 g sodium dodecyl benzene sulfonate white powder was mixed with 96.52 mL of the distilled water, and the mixture was stirred until it was completely dissolved, to obtain a 1.0% aqueous solution of sodium dodecyl benzene sulfonate as an emulsifier. Then, the 12.5 g of Dulux waterborne acrylic resin was added to 97.0 mL of the 1.0% sodium dodecyl benzene sulfonate aqueous solution, and the mixed solution was then put in a 60 °C constant-temperature water bath and stirred at 1200 rpm for 30 min to gain the stable core material emulsion. The 1–2 drops of octanol were added for defoaming. The third step is microencapsulation: the urea-formaldehyde prepolymer was dropped into the prepared core material at the speed of 300 rpm, and then the citric acid was added gradually to adjust the pH to 2.5–3.0. The temperature was slowly raised to 50 °C and held for 3 h. Finally, the obtained product was filtered by suction and distilled water was added to rinse off the excess emulsifier. Then, the product was heated and dried at 80 °C for 4 h, and the white powder obtained was the sample 1# of the orthogonal test (Table 2). The specific preparation progress of samples 2–4# is the same as that of sample 1#.

The most important factors affecting the performance of microcapsules were determined by the above orthogonal experiments. On this basis, the single-factor independent experiment was carried out to determine the optimal core wall ratio of microcapsules and further optimize the preparation scheme. In the single-factor independent experiment, the water bath temperature was set at 70 °C, the aging time was 5 d, and the core–wall ratio was set as 0.42:1, 0.50:1, 0.58:1, 0.67:1, 0.75:1, 0.83:1 and 0.92:1 (samples 5#−11#), respectively.

### 2.3. Preparation of Coatings

First, the 0.4 g microcapsules with 0.58:1 of core–wall ratio were added to 3.6 g waterborne acrylic resin coatings, and the mixture was evenly mixed to form a waterborne coating with 10.0% microcapsule concentration. The prepared paint was coated on the surface of the Tilia europaea panels using a SZQ tetrahedral fabricator (Jinghai Science and Technology Testing Machinery Factory, Tianjin, China), dried at room temperature for 30 min, then polished with 800 mesh sandpaper and wiped with a dry cloth. The above process was repeated three times, and the dry coating thickness was about 60 μm. 

### 2.4. Testing and Characterization

A ZEISS electron microscope AX10 (Carl Zeiss AG, Aalen, Germany) and a Quanta 200 environment scanning electron microscope (SEM), FEI Company, Hillsboro, OR, USA were used to characterize the microcapsule morphology [25]. A VERTEX 80 V infrared spectrum analyzer (Germany Bruker Co., Ltd., Karlsruhe, Germany) was used to analyze the chemical composition of microcapsules [26]. The encapsulation ratio of microcapsules can be tested as follows: the 1.0 g microcapsules (m_1_) were fully ground in a mortar and placed in a sand core funnel. Then, a certain amount of ethyl acetate was added, the microcapsules were fully soaked for 72 h, and the ethyl acetate was changed every 24 h. The mixture was rinsed and filtered with deionized water, and the product was dried and weighed to obtain the residual wall mass (m_2_). The encapsulation ratio (c) can be calculated by Formula (1) [27].

c = (m_1_ − m_2_)/m_1_ × 100% (1)

The gloss of waterborne coatings was tested by HG268 gloss meter produced by 3NH Technology Co., Ltd., Shenzhen, China. The light incident angle of 60° was used. The hardness of paint film was measured by pencil hardness tester with a 6H-6B pencil. The pencil was pushed forward at an angle to the film. When the coating was not damaged, the maximum hardness of the pencil was the hardness of the paint film. The adhesion of the paint film was tested by QFH-HG600 film scriber (Tianjin Jingke Material Experiment Machine Factory, Tianjin, China), and the adhesion grade of the paint film was judged by the degree of damage. There are six grades: 0, 1, 2, 3, 4 and 5, of which grade 0 has the best adhesion and grade 5 has the worst adhesion. The impact resistance of paint film was measured by a QCJ film impactor tester (Tianjin Jingkelian Material Testing Machine Co., Ltd., Tianjin, China). The Model AG-IC100KN precision electronic universal capability experiment machine (Shimadzu Co., Ltd., Kyoto, Japan) was used to measure the elongation at break of the coating. The paint film was coated on the glass substrate and peeled off. After being stripped, the coatings were made into thin sheets, and then both ends of the coatings were fixed with clamps to prevent it from sliding. The coatings were destroyed at a tensile speed of 0.12 mm/min and a certain longitudinal load. The elongation at break was expressed by the ratio of the displacement value of the coating at break to the original length of the coating. Thermogravimetric analysis (STA8000) (PerkinElmer Inc., Waltham, MA, USA) was used to determine the thermal stability of the microcapsules at a heating rate of 20 °C·min^−1^ in a nitrogen atmosphere. The orthogonal design assistant version 3.1 was used to analyze the data of orthogonal experiment. The orthogonal design assistant version 3.1 is a professional software for orthogonal experimental design and analysis of experimental results. All the above measurements were repeated at least four times with the error lower than 5.0%.

## 3. Results and Discussion

### 3.1. Orthogonal Experiment Analysis

#### 3.1.1. Characterization of Microcapsule Morphology

**Figure 1:**
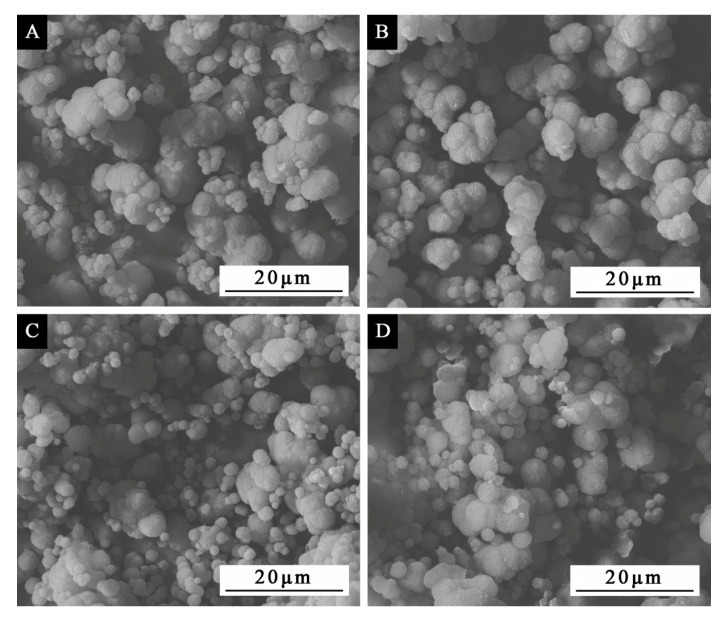
SEM morphologies of microcapsules by orthogonal experiment: samples 1–4# in Table 2.

The morphology of orthogonal sample microcapsules is shown in Figure 1. The particle size for sample 1# (Figure 1A) is not uniform, about 3–8 μm. The microcapsules are well coated with a small amount of agglomeration. Sample 2# (Figure 1B) shows that the size of microcapsules is about 5 μm, the particles agglomerate less and the microcapsules are well-coated. The particle size of sample 3# (Figure 1C) is about 3–8 μm, which is uneven and has a certain degree of agglomeration. The particle size of sample 4# (Figure 1D) is not uniform, about 3–8 μm, and the particles are partially precipitated, accompanied by agglomeration. By comparing the morphology of the four samples in the orthogonal test, it can be seen that the morphology of sample 2# was relatively good, with the slightly rough surface and the uniform particle size, followed by sample 3#. The results showed that urea-formaldehyde-coated waterborne acrylic microcapsules with a particle size of about 5 μm were successfully produced.

#### 3.1.2. Analysis of the Chemical Composition of Microcapsules

**Figure 2:**
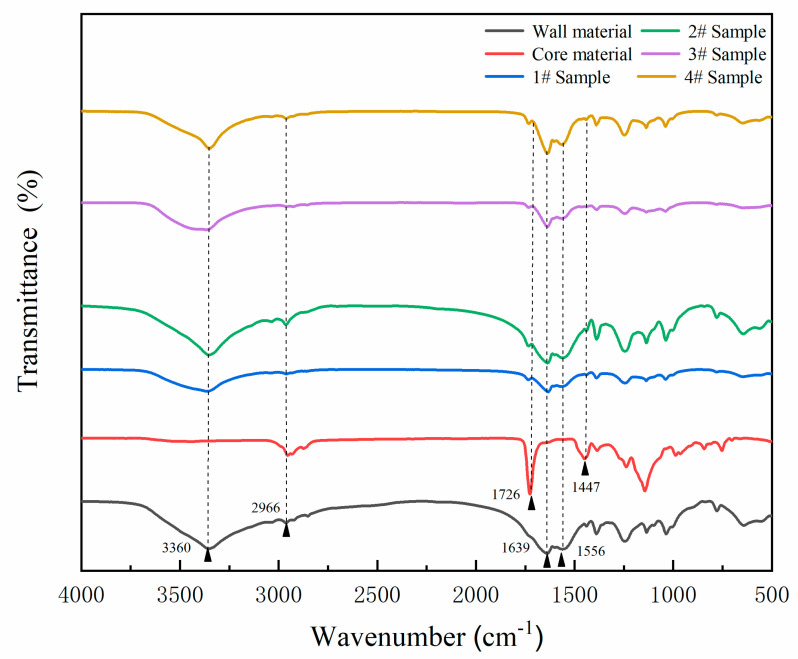
The infrared spectrum of wall material, core material and samples 1–4# of microcapsule.

Figure 2 is the FTIR of wall-material urea-formaldehyde resin, core material of Dulux waterborne acrylic resin, and microcapsules (samples 1–4# in Table 2). The absorption at 3360 cm^−1^ was attributed to the N–H stretching vibration, which was the characteristic absorption of the urea-formaldehyde resin. The special absorption at 1556 cm^−1^ was assigned to C–N, and the absorption at 1639 cm^−1^ was due to the stretching vibration of C=O. Corresponding peaks also appeared in the infrared spectrum of the microcapsules (samples 1–4# in Table 2), and it was determined that the corresponding urea-formaldehyde resin wall material was generated in the prepared microcapsules. The absorption at 2966 and 1447 cm^−1^ was assigned to C–H. The characteristic absorption at 1726 cm^−1^ was assigned to C=O in waterborne acrylic resin. The presence of waterborne acrylic resin in the microcapsules was proved in Figure 2, and the ingredients were not destroyed. Therefore, it was proved that urea-formaldehyde-coated Dulux waterborne acrylic resin microcapsules were successfully prepared.

#### 3.1.3. Yield Analysis of Microcapsules

The yield of microcapsules is a significant indicator for determining the quality of the prepared microcapsules. It is of great significance to produce high-yield microcapsules under the condition of less drug consumption in industrial production applications [28]. The samples 1–4# prepared in the orthogonal test were weighed separately to obtain the output of each sample, and the range (R) results are shown in Table 3. Among them, sample 4# had the largest output at 32.75 g. Variance results are shown in Table 4. Among the three factors, the core–wall ratio was the most influential factor on the microcapsule output results, followed by the water bath temperature. According to the output results alone, it can be concluded that the preferred preparation process of urea-formaldehyde coated waterborne acrylic microcapsules was 0.67:1 of the core–wall ratio, 70 °C of the water bath temperature and 5 d of depositing time. However, from the perspective of microcapsule morphology, the microscopic characteristics of sample 2# were the best, so the better preparation process parameters of microcapsules must be determined in conjunction with analysis of other results.

#### 3.1.4. Encapsulation Ratio Analysis of Microcapsules

The encapsulation ratio of microcapsules is an important indicator to determine the repair effect of microcapsules. The encapsulation ratio refers to the content of the core material as a repair agent coated in the wall material. The core material may be difficult to be successfully coated due to factors such as cracking of the wall material or excessive agglomeration during the preparation process, so the content of the coated core material has an important influence on the repair effect [29]. The range and variance of the encapsulation ratio of the microcapsules in the orthogonal test are shown in Table 5 and Table 6. The factor that had the greatest impact on the encapsulation ratio of microcapsule was the core–wall ratio, followed by the depositing time. The sample 3# had the highest encapsulation ratio of 39.0%. According to the encapsulation ratio results, it can be concluded that the core–wall ratio of 0.67:1 and the depositing time of 5 d were the preferred processes for preparing urea-formaldehyde-coated waterborne acrylic microcapsules, and the water bath temperature had basically no effect on the encapsulation ratio.

The results showed that among the three factors, the core–wall ratio was the factor that had the greatest influence on the yield and encapsulation ratio of microcapsules in the orthogonal experiment. In order to prepare microcapsules with a good morphology and excellent performance, a single-factor independent test for the core–wall ratio was carried out, based on the orthogonal test results. The 70 °C of the water bath temperature and 5 d of depositing time were the best levels in the analysis of yield results, and 5 d depositing time was the better level in the analysis of the encapsulation ratio results. Considering that the microscopic morphology of sample 2# was the best, the 70 °C of the water bath temperature and 5 d of the depositing time were selected. On this basis, a single-factor test was conducted for the core–wall ratio to analyze its effect on the performance of microcapsules.

### 3.2. Single Factor Experiment Analysis

#### 3.2.1. Characterization of Microcapsule Morphology

**Figure 3:**
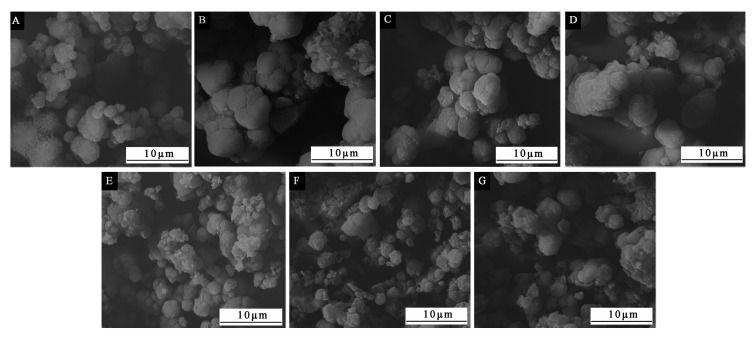
The SEM morphologies of microcapsules with different core–wall ratios: (**A**) 0.42:1, (**B**) 0.50:1, (**C**) 0.58:1, (**D**) 0.67:1, (**E**) 0.75:1, (**F**) 0.83:1, (**G**) 0.92:1.

The SEM morphologies of the microcapsules by the single factor test of the core–wall ratio are shown in Figure 3. Comparing samples with different core–wall ratios, it was obvious that as the core–wall ratio increased, the agglomeration and precipitation of the microcapsules also became serious. Microcapsules with core–wall ratios of 0.75:1, 0.83:1 and 0.92:1 (Figure 3E–G) can be observed with obvious flocs, and microcapsules with a core–wall ratio of 0.58:1 had a larger particle size than other microcapsules, but less agglomeration and better morphology. Judging from the overall effect in Figure 3, the microcapsule morphology was relatively better when the core–wall was relatively small.

#### 3.2.2. Analysis of the Chemical Composition of Microcapsules

**Figure 4:**
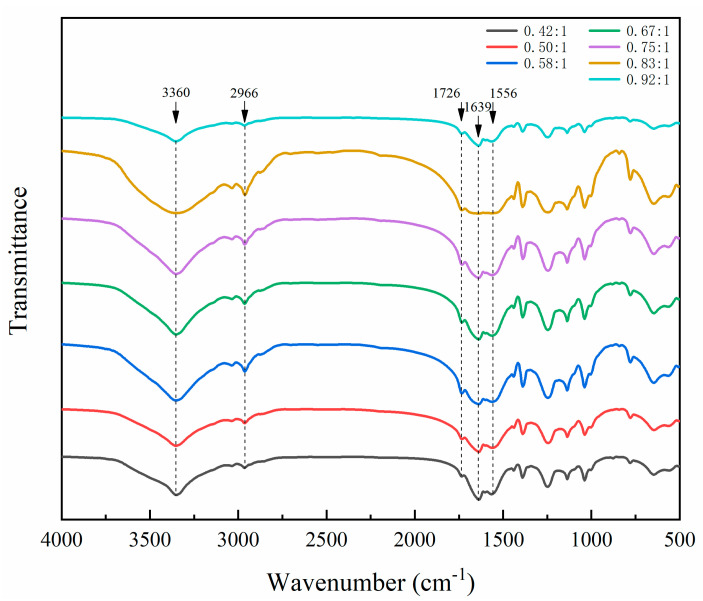
FTIR spectrum of microcapsules with different core–wall ratios.

The FTIR of microcapsules by single-factor experiment is shown in Figure 4. The characteristic absorption at 3360 and 1556 cm^−1^ was assigned to N–H and C–N. The absorption at 1639 cm^−1^ was attributed to the C=O stretching vibration of urea-formaldehyde resin. The characteristic absorption at 2966 and 1447 cm^−1^ was assigned to C–H. The peak at 1726 cm^−1^ was the characteristic absorption of C=O in waterborne acrylic resin. It can be found in Figure 4 that the characteristic absorption of microcapsules with different core–wall ratios was consistent. The microcapsules were successfully prepared, and the chemical composition of microcapsules with different core–wall ratios had not changed.

#### 3.2.3. Yield Analysis of Microcapsules

The microcapsules prepared by the single-factor experiment were weighed separately, and the obtained yield results are shown in Table 7. It can be clearly found that as the core–wall ratio continued to increase, the yield of microcapsules also continued to increase, from 28.27 to 32.85 g. As the quality of the wall material remained unchanged, the quality of the core material continued to increase, which means that the content of the repairable agent that can be coated increased, resulting in an increase in the yield of microcapsules. The yield of microcapsules with a 0.92:1 core–wall ratio was the largest, but the overall yield of the seven samples was not too obvious.

#### 3.2.4. Encapsulation Ratio Analysis of Microcapsules

The encapsulation ratio results of microcapsules with different core–wall ratios are shown in Table 8. With the increase in the core–wall ratio (the increase in the core material quality), the encapsulation ratio of microcapsules basically tended to increase first and then decrease. When the core–wall ratio raised from 0.42:1 to 0.67:1, the encapsulation ratio increased from 33.0% to 41.0%, but as the core–wall ratio continued to increase to 0.92:1, the encapsulation ratio decreased to 24.0%. Due to the increase in the quality of the core material, the content of the core material successfully coated by the wall material improved, which raised the encapsulation ratio to a certain extent. However, combining with the microscopic morphology of microcapsules with different core–wall ratios in Figure 3, it can be found that when the quality of core material was too high, the agglomeration of the prepared microcapsules was serious. The core material cannot be completely coated successfully, resulting in a reduction in the encapsulation ratio of microcapsules. The encapsulation ratio of samples in the single factor experiment was not too high. This may be because the selected repair agent was waterborne acrylic resin. The presence of water and additives would affect the final microcapsule formation and reduce the microcapsules performance. From Table 8, when the core–wall ratio was 0.58:1 and 0.67:1, the encapsulation ratio of microcapsules was relatively high.

### 3.3. Effect of Depositing Time on the Morphology of Microcapsules

**Figure 5:**
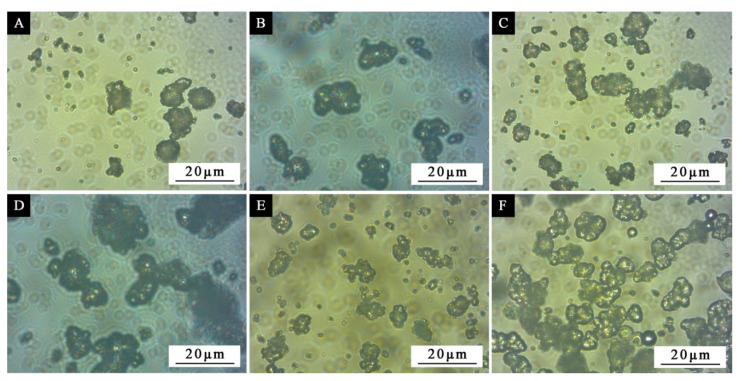
The optical microscopy morphologies of microcapsules prepared under different depositing time: (**A**) 0, (**B**) 1 d, (**C**) 2 d, (**D**) 3 d, (**E**) 4 d, (**F**) 5 d.

The results of the encapsulation ratio can better characterize the performance of microcapsules than the results of yield [30], and the orthogonal test results of the encapsulation ratio showed that the depositing time had a certain influence on the encapsulation ratio of microcapsules. In order to understand the formation mechanism of microcapsules and investigate the influence of microcapsules on waterborne wood coatings, according to the above results, the microcapsules of the 0.58:1 core–wall ratio with better overall performance were selected to be placed in 0, 1, 2, 3, 4 and 5 d. The formation state of the microcapsules after suction filtration under different depositing times was observed (Figure 5). As the depositing time increased, it can be seen that the observed spherical particles would increase. Without aging for a certain period of time, the microcapsules (Figure 5A) had fewer particles and obvious dispersion after direct filtration and drying. The particles of microcapsules (Figure 5B) that had been aged for 1 d were bound to each other. With the extension of the depositing time, the bonding particles became more and more and even agglomerated. When the depositing time was 5 d (Figure 5F), it can be clearly seen that the particles partially gathered together. It showed that the formation state of microcapsules had a certain relationship with the depositing time.

**Figure 6:**
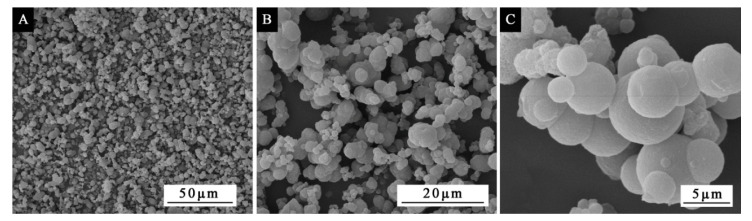
The SEM morphology of microcapsule prepared under two months of deposition: (**A**) low magnification, (**B**) medium magnification, (**C**) high magnification.

In order to more clearly comprehend the effect of depositing time on the morphology of microcapsules, the microcapsules with 0.58:1 of the core–wall ratio were prepared under two months of deposition. The SEM morphology of microcapsule at different time is shown in Figure 6. After a long period of aging, the microcapsules were concentrated and bound together, with a small amount of agglomeration, but there was no particularly serious agglomeration precipitation phenomenon. Compared with the microcapsules with a larger core–wall ratio in the single factor experiment, the morphology of Figure 6 was better. It proved that although the microcapsule particles would gradually increase with the rise in depositing time, accompanied by the phenomenon of adhesion, it would not lead to the microcapsules to completely deposit and reunite due to the too-long depositing time. At the same time, it also showed that the core–wall ratio was a more important factor that affected the morphology of the microcapsules relative to depositing time.

### 3.4. Effect of Microcapsules on Properties of Waterborne Coatings

The gloss, hardness, adhesion, impact resistance and elongation at break in waterborne coatings with 0 and 10.0% of waterborne acrylic microcapsules (core–wall ratio of 0.58:1) are shown in Table 9. It can be found that the gloss of the waterborne coating film after adding waterborne acrylic microcapsules was lower than that of the paint film without microcapsules. That was because the addition of microcapsules enhanced the surface roughness of the paint film, and the increase in particles on the surface led to the enhancement of the diffuse reflection of the coating surface, thus reducing the gloss of the paint film. Meanwhile, with the addition of waterborne acrylic microcapsules, the hardness of the film rose from HB to 2H, the adhesion declined from 0 to 1 grade, and the impact resistance rose from 5.0 to 15.0 kg·cm. It can be seen that although the waterborne acrylic microcapsule added into the waterborne coating had some impact on the performance of the coating, in the case of 10.0% microcapsule, the performance of the coating did not significantly decline; on the contrary, the impact resistance of the coating was also improved. According to the results of elongation at break test, the elongation at break of the coating increased obviously after adding the microcapsule to the waterborne coating. This was mainly because the presence of 10.0% microcapsules enhanced the toughness of the coating, making the paint film difficult to break.

**Figure 7:**
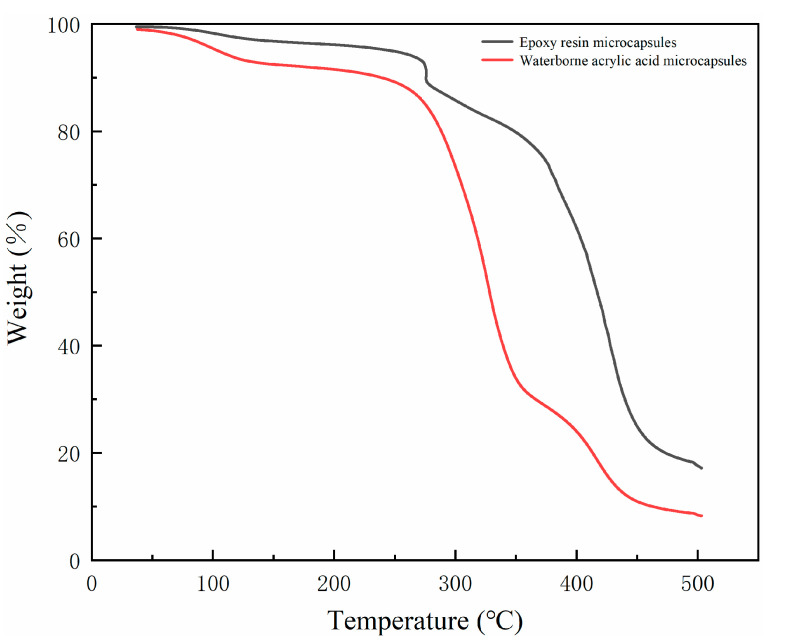
Thermogravimetry of waterborne acrylic microcapsules and epoxy microcapsules.

The coating was compared with the performance of the waterborne coating with epoxy resin microcapsules [31]. According to Table 9, it can be found that the gloss of the two kinds of coating with microcapsules was similar, but the hardness and impact resistance of the coating with epoxy resin microcapsules were relatively high, while the adhesion was low. In Table 9, the core–wall ratio of epoxy resin microcapsule is 0.83:1, which is higher than that of waterborne acrylic microcapsules (0.58:1), and the hardness of waterborne acrylic is relatively low [32]. Therefore, the hardness of the coating prepared by epoxy resin microcapsules is higher than that of waterborne acrylic microcapsules. The results showed that the coating with epoxy resin had better elongation at break than that with waterborne acrylic microcapsule, which may be due to the fact that the flexibility of waterborne acrylic resin is not as good as that of epoxy resin, so the elongation at break was low. As shown in thermogravimetric curves (Figure 7) and thermal properties (Table 10), 5% and 10% thermal decomposition temperatures (T_5%_ and T_10%_, respectively) of waterborne acrylic microcapsules were 104.4 and 239.34 °C, whereas the epoxy resin microcapsules showed highly increased values of 248.05 and 275.76 °C. The epoxy resin improves thermo-stability of microcapsules. However, compared with epoxy resin, the waterborne acrylic resin can be cured at room temperature without heating and has lower requirements for temperature and stronger practicability.

## 4. Conclusions

The influences of the three factors (core–wall ratio, water bath temperature and depositing time) on the yield, encapsulation ratio, particle size and morphology of microcapsules are explored to determine the preferred level of 70 °C water bath temperature and 5 d depositing time. Among the three factors, the core–wall ratio is the factor that has the greatest impact on the performance of the microcapsule. In the single-factor test with the core–wall ratio as a variable, when the core–wall ratio is relatively small, the microcapsules have good morphological characteristics. The microcapsules with 0.58:1 of the core–wall ratio have a uniform and obvious particle size. When the core–wall ratio is relatively large, the yield of the microcapsules is higher. When the core–wall ratio is 0.58:1 or 0.67:1, it has a better encapsulation ratio. In short, the overall performance of microcapsules with 0.58:1 of the core–wall ratio is better in morphology, yield and encapsulation ratio. The microcapsules would not be completely deposited and agglomerated due to their long depositing time. When the waterborne acrylic microcapsules with 0.58:1 of the core–wall ratio were added into the waterborne coating with 10.0% concentration, the microcapsules have a good effect on the film properties, and the elongation at break of the film is significantly improved. The results provide a technical reference for improving the toughness of waterborne coatings cured at room temperature.

## Figures and Tables

**Table 1:** Orthogonal experimental arrangements.

Sample	Core–Wall Ratio	Water Bath Temperature (°C)	Depositing Time (d)
1#	0.42:1	50	1
2#	0.42:1	70	5
3#	0.67:1	50	5
4#	0.67:1	70	1

**Table 2:** The amount of substance in the orthogonal experiment.

Sample	Urea (g)	37.0% Formaldehyde (g)	Waterborne Acrylic Resin (g)	Sodium Dodecyl Benzene Sulfonate (g)	Deionized Water (mL)
1#	20.0	27.0	12.5	0.975	96.52
2#	20.0	27.0	12.5	0.975	96.52
3#	20.0	27.0	20.0	1.56	154.44
4#	20.0	27.0	20.0	1.56	154.44

**Table 3:** Range results of microcapsule yield.

Sample	Core–Wall Ratio	Water Bath Temperature (°C)	Depositing Time (d)	Yield (g)
1#	0.42:1	50	1	26.07
2#	0.42:1	70	5	28.44
3#	0.67:1	50	5	31.08
4#	0.67:1	70	1	32.75
Mean 1	27.255	28.575	29.410	
Mean 2	31.915	30.595	29.760	
R	4.660	2.020	0.350	

**Table 4:** Variance results of microcapsule yield.

Factor	Sum of Squared Deviations	Degrees of Freedom	F Ratio	F Critical Value	Significance
Core–Wall Ratio	27.716	1	178.000	161.000	*
Water Bath Temperature (°C)	4.080	1	33.443	161.000	
Depositing Time (d)	0.122	1	1.000	161.000	
Error	0.12	1			

**Table 5:** Range results of microcapsule encapsulation ratio.

Sample	Core–Wall Ratio	Water Bath Temperature (°C)	Depositing Time (d)	Encapsulation Ratio (%)
1#	0.42:1	50	1	33.0
2#	0.42:1	70	5	35.0
3#	0.67:1	50	5	39.0
4#	0.67:1	70	1	37.0
Mean 1	34.000	36.000	35.000	
Mean 2	38.000	36.000	37.000	
R	4.000	0	2.000	

**Table 6:** Variance results of microcapsule encapsulation ratio.

Factor	Sum of Squared Deviations	Degrees of Freedom	F Ratio	F Critical Value	Significance
Core–wall ratio	16.000	1	4.000	161.000	
Water bath temperature (°C)	0	1	0	161.000	
Depositing time (d)	4.000	1	1.000	161.000	
Error	4.00	1			

**Table 7:** Yield results of microcapsules in the single factor experiment.

Sample	Core–Wall Ratio	Core Material Quality (g)	Yield (g)
5#	0.42:1	12.5	28.27
6#	0.50:1	15.0	29.48
7#	0.58:1	17.5	30.79
8#	0.67:1	20.0	31.80
9#	0.75:1	22.5	32.18
10#	0.83:1	25.0	32.35
11#	0.92:1	27.5	32.85

**Table 8:** Encapsulation ratio results of microcapsules in the single factor experiment.

Sample	Core–Wall Ratio	Core Material Quality (g)	Encapsulation Ratio (%)
5#	0.42:1	12.5	33.0
6#	0.50:1	15.0	34.0
7#	0.58:1	17.5	39.0
8#	0.67:1	20.0	41.0
9#	0.75:1	22.5	30.0
10#	0.83:1	25.0	31.0
11#	0.92:1	27.5	24.0

**Table 9:** Performance comparison of waterborne coatings before and after adding microcapsules.

Core Material	Optimum Core–Wall Ratio	Microcapsules Concentration (%)	Gloss (%)	Hardness	Adhesion (Grade)	Impact Resistance (kg·cm)	Elongation at Break (%)
-	-	0	27.90	HB	0	5.0	5.25
Waterborne Acrylic Acid	0.58:1	10.0	5.10	2H	1	15.0	16.69
Epoxy Resin	0.83:1	10.0	5.0	5H	3	20.0	35.00

**Table 10:** Thermal properties of waterborne acrylic acid microcapsules and epoxy resin microcapsules.

Core Material	T_5%_ (°C)	T_10%_ (°C)
Waterborne Acrylic Acid	104.4	239.34
Epoxy Resin	248.05	275.76
